# *N*-(2-Chloro­phen­yl)-2-(4,6-dimethyl­pyrimidin-2-ylsulfan­yl)acetamide

**DOI:** 10.1107/S1600536809011520

**Published:** 2009-04-02

**Authors:** Qiang Li, Wei Wang, Hui Wang, Yan Gao, Hong Qiu

**Affiliations:** a School of Chemical Engineering, University of Science and Technology, Liaoning Anshan 114051, People’s Republic of China; b Hermann Gmeiner Vocational Technical College, Qiqihar University, Heilongjiang, Qiqihar 161006, People’s Republic of China

## Abstract

In the title compound, C_14_H_14_ClN_3_OS, the 4,6-dimethyl­pyrimidine ring and the chloro­benzene ring subtend a dihedral angle of 80.0 (2)°. The length of the C*sp* ^2^—S bond is significantly shorter than that of the C*sp* ^3^—S bond. The crystal structure is stabilized by inter­molecular N—H⋯O, C—H⋯O and C—H⋯N hydrogen bonding, and C—H⋯π inter­actions.

## Related literature

For bond-length data, see: Gao *et al.* (2007 ▶). For heteroatom-rich compounds as effective precursors for active mol­ecules, see: Huynh *et al.* (2005 ▶); Ye *et al.* (2006 ▶).
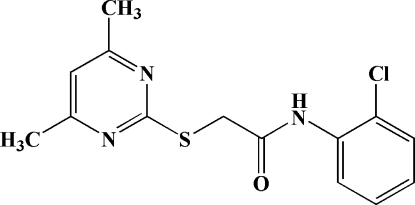


## Experimental

### 

#### Crystal data

C_14_H_14_ClN_3_OS
*M* *_r_* = 307.79
Orthorhombic, 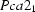

*a* = 26.494 (5) Å
*b* = 4.6736 (9) Å
*c* = 11.931 (2) Å
*V* = 1477.3 (5) Å^3^
*Z* = 4
Mo *K*α radiation
μ = 0.40 mm^−1^
*T* = 113 K
0.30 × 0.26 × 0.20 mm

#### Data collection

Rigaku Saturn diffractometer
Absorption correction: multi-scan (*SADABS*; Sheldrick, 1996 ▶) *T*_min_ = 0.890, *T*_max_ = 0.925
8870 measured reflections
2573 independent reflections
2445 reflections with *I* > 2σ(*I*)
*R*_int_ = 0.031

#### Refinement

*R*[*F* ^2^ > 2σ(*F* ^2^)] = 0.024
*wR*(*F* ^2^) = 0.061
*S* = 1.07
2573 reflections
187 parameters
2 restraints
H atoms treated by a mixture of independent and constrained refinement
Δρ_max_ = 0.17 e Å^−3^
Δρ_min_ = −0.20 e Å^−3^
Absolute structure: Flack (1983 ▶), 1199 Freidel pairs
Flack parameter: 0.00 (5)

### 

Data collection: *CrystalClear* (Molecular Structure Corporation & Rigaku, 1999 ▶); cell refinement: *CrystalClear* ; data reduction: *CrystalClear*; program(s) used to solve structure: *SHELXS97* (Sheldrick, 2008 ▶); program(s) used to refine structure: *SHELXL97* (Sheldrick, 2008 ▶); molecular graphics: *SHELXTL* (Sheldrick, 2008 ▶); software used to prepare material for publication: *SHELXTL*.

## Supplementary Material

Crystal structure: contains datablocks global, I. DOI: 10.1107/S1600536809011520/at2755sup1.cif

Structure factors: contains datablocks I. DOI: 10.1107/S1600536809011520/at2755Isup2.hkl

Additional supplementary materials:  crystallographic information; 3D view; checkCIF report

## Figures and Tables

**Table 1:** Hydrogen-bond geometry (Å, °)

*D*—H⋯*A*	*D*—H	H⋯*A*	*D*⋯*A*	*D*—H⋯*A*
N1—H1⋯O1^i^	0.873 (11)	2.054 (12)	2.8414 (18)	149.6 (18)
C2—H2⋯O1^ii^	0.93	2.46	3.213 (2)	138
C8—H8*A*⋯*Cg*1^i^	0.97	2.92	3.832 (2)	157
C13—H13*B*⋯*Cg*1^iii^	0.96	2.99	3.592 (2)	122

Symmetry codes: (i) 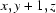; (ii) 
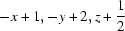
; (iii) 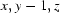. *Cg*1 is the centroid of the N2/N3/C9–C12 ring.

**Fig. 1.**
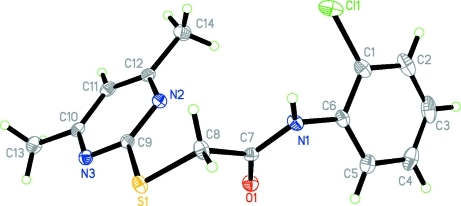
View of the molecule of (I) showing the atom-labeling scheme. Displacement ellipsoids are drawn at the 35% probability level.

## supplementary crystallographic information

### Comment

The heteroatom-rich compounds have been intensively studied due to their applications including effective precursors for active molecule (Ye *et al.*, 2006; Huynh *et al.*, 2005). Now, we have synthesized the title compound, (I), from 4,6-dimethylpyrimidin-2-thiol with 2-chlorophenyl carbamic chloride. Here we report the crystal structure determination of the title compound.

The molecular structure of (I) and the atom-numbering scheme are shown in Fig. 1. The molecule contains a benzene ring and a pyrimidine ring. The dihedral angle between the benzene ring and benzo[*d*]thiazole ring is 80.0 (2)°, which indicate the two rings are close to be perpendicular. Cl atom attached to the benzene ring is coplanar to the benzene ring with an r.m.s deviation of 0.0130 (3) Å. The deviations with the pyrimidine ring plane of C13 and C14 atoms are 0.0544 (3) and 0.0005 (3) Å, respectively. The C6—N1—C7—C8 torsion angle of 177.61 (15)° indicates that the acylamide group are nearly coplanar with the benzene ring plane. As a result of π-π conjugation, the C*sp*^2^—S bond [S1—C9 = 1.7646 (17) Å] is significantly shorter than the C*sp*^3^—S bond [S1—C8 = 1.7947 (17) Å]. These values compare with the values of 1.772 (3) and 1.801 (2) Å reported in the literature (Gao *et al.*, 2007).

The crystal structure is stabilized by inter molecular C—H···O and C—H···N hydrogen bonding, and C—H···π interactions (Table 1).

### Experimental

The title compound was synthesized by the reaction of from the 4,6-dimethylpyrimidin-2-thiol with 2-chlorophenyl carbamic chloride in the refluxing ethanol. Crystals of (I) suitable for single-crystal X-ray analysis were grown by slow evaporation of a solution in chloroform/acetone.

### Refinement

The H atoms attached to N atom was located in a different density map and the atomic coordinates allowed to refine freely. Other H atoms were positioned geometrically and refined as riding (C—H = 0.93–0.97 Å) and allowed to ride on their parent atoms, with *U*_iso_(H) =1.2*U*_eq_(parent) or 1.5*U*_eq_(parent).

### Crystal data

**Table:**

C_14_H_14_ClN_3_OS	*F*(000) = 640
*M**_r_* = 307.79	*D*_x_ = 1.384 Mg m^−^^3^
Orthorhombic, *P**c**a*2_1_	Mo *K*α radiation, λ = 0.71073 Å
Hall symbol: P 2c -2ac	Cell parameters from 4682 reflections
*a* = 26.494 (5) Å	θ = 1.5–27.9°
*b* = 4.6736 (9) Å	µ = 0.40 mm^−^^1^
*c* = 11.931 (2) Å	*T* = 113 K
*V* = 1477.3 (5) Å^3^	Prism, colourless
*Z* = 4	0.30 × 0.26 × 0.20 mm

### Data collection

**Table:**

Rigaku Saturn diffractometer	2573 independent reflections
Radiation source: rotating anode	2445 reflections with *I* > 2σ(*I*)
confocal	*R*_int_ = 0.031
ω scans	θ_max_ = 25.0°, θ_min_ = 1.5°
Absorption correction: multi-scan (*SADABS*; Sheldrick, 1996)	*h* = −23→31
*T*_min_ = 0.890, *T*_max_ = 0.925	*k* = −5→5
8870 measured reflections	*l* = −14→14

### Refinement

**Table:**

Refinement on *F*^2^	Secondary atom site location: difference Fourier map
Least-squares matrix: full	Hydrogen site location: inferred from neighbouring sites
*R*[*F*^2^ > 2σ(*F*^2^)] = 0.024	H atoms treated by a mixture of independent and constrained refinement
*wR*(*F*^2^) = 0.061	*w* = 1/[σ^2^(*F*_o_^2^) + (0.0391*P*)^2^] where *P* = (*F*_o_^2^ + 2*F*_c_^2^)/3
*S* = 1.07	(Δ/σ)_max_ = 0.001
2573 reflections	Δρ_max_ = 0.17 e Å^−^^3^
187 parameters	Δρ_min_ = −0.20 e Å^−^^3^
2 restraints	Absolute structure: Flack (1983), 1199 Freidel pairs
Primary atom site location: structure-invariant direct methods	Flack parameter: 0.00 (5)

### Special details

**Table:**

Geometry. All e.s.d.'s (except the e.s.d. in the dihedral angle between two l.s. planes) are estimated using the full covariance matrix. The cell e.s.d.'s are taken into account individually in the estimation of e.s.d.'s in distances, angles and torsion angles; correlations between e.s.d.'s in cell parameters are only used when they are defined by crystal symmetry. An approximate (isotropic) treatment of cell e.s.d.'s is used for estimating e.s.d.'s involving l.s. planes.
Refinement. Refinement of *F*^2^ against ALL reflections. The weighted *R*-factor *wR* and goodness of fit *S* are based on *F*^2^, conventional *R*-factors *R* are based on *F*, with *F* set to zero for negative *F*^2^. The threshold expression of *F*^2^ > σ(*F*^2^) is used only for calculating *R*-factors(gt) *etc*. and is not relevant to the choice of reflections for refinement. *R*-factors based on *F*^2^ are statistically about twice as large as those based on *F*, and *R*- factors based on ALL data will be even larger.

### Fractional atomic coordinates and isotropic or equivalent isotropic displacement parameters (Å^2^)

**Table:**

	*x*	*y*	*z*	*U*_iso_*/*U*_eq_	
S1	0.689926 (15)	1.07672 (8)	0.28992 (4)	0.02168 (11)	
Cl1	0.53054 (2)	1.55461 (10)	0.63460 (4)	0.03848 (14)	
O1	0.58637 (4)	0.8871 (2)	0.35668 (11)	0.0216 (3)	
N1	0.55577 (5)	1.3204 (3)	0.40980 (12)	0.0193 (3)	
N2	0.67886 (5)	1.0007 (3)	0.51049 (13)	0.0216 (3)	
N3	0.74415 (5)	0.7412 (3)	0.41469 (12)	0.0198 (3)	
C1	0.49282 (7)	1.3208 (4)	0.55761 (16)	0.0250 (4)	
C2	0.44695 (7)	1.2326 (4)	0.60204 (17)	0.0353 (5)	
H2	0.4361	1.3025	0.6710	0.042*	
C3	0.41781 (7)	1.0427 (5)	0.5439 (2)	0.0403 (6)	
H3	0.3873	0.9816	0.5740	0.048*	
C4	0.43334 (7)	0.9406 (4)	0.44072 (19)	0.0350 (5)	
H4	0.4134	0.8106	0.4017	0.042*	
C5	0.47894 (6)	1.0334 (4)	0.39558 (18)	0.0254 (4)	
H5	0.4891	0.9674	0.3256	0.031*	
C6	0.50924 (6)	1.2233 (3)	0.45406 (14)	0.0204 (4)	
C7	0.59151 (6)	1.1465 (3)	0.36648 (14)	0.0171 (3)	
C8	0.63851 (6)	1.3054 (4)	0.32946 (15)	0.0233 (4)	
H8A	0.6495	1.4287	0.3901	0.028*	
H8B	0.6300	1.4267	0.2663	0.028*	
C9	0.70557 (6)	0.9262 (3)	0.42103 (14)	0.0179 (3)	
C10	0.75680 (6)	0.6156 (3)	0.51203 (15)	0.0201 (4)	
C11	0.73123 (6)	0.6745 (4)	0.61057 (15)	0.0238 (4)	
H11	0.7400	0.5841	0.6772	0.029*	
C12	0.69218 (6)	0.8721 (4)	0.60723 (16)	0.0231 (4)	
C13	0.80089 (7)	0.4138 (4)	0.50769 (18)	0.0280 (4)	
H13A	0.8046	0.3412	0.4329	0.042*	
H13B	0.7952	0.2576	0.5584	0.042*	
H13C	0.8311	0.5135	0.5291	0.042*	
C14	0.66199 (8)	0.9520 (5)	0.70853 (18)	0.0363 (5)	
H14A	0.6604	1.1567	0.7146	0.054*	
H14B	0.6778	0.8745	0.7743	0.054*	
H14C	0.6285	0.8762	0.7016	0.054*	
H1	0.5635 (7)	1.500 (2)	0.4204 (17)	0.027 (5)*	

### Atomic displacement parameters (Å^2^)

**Table:**

	*U*^11^	*U*^22^	*U*^33^	*U*^12^	*U*^13^	*U*^23^
S1	0.02112 (19)	0.0217 (2)	0.0222 (2)	0.00522 (16)	0.00482 (17)	0.0057 (2)
Cl1	0.0551 (3)	0.0323 (3)	0.0280 (3)	0.0023 (2)	0.0035 (2)	−0.0090 (2)
O1	0.0241 (6)	0.0138 (6)	0.0270 (7)	0.0011 (5)	0.0005 (5)	−0.0002 (5)
N1	0.0224 (7)	0.0105 (7)	0.0249 (8)	−0.0024 (5)	0.0059 (6)	−0.0004 (6)
N2	0.0201 (7)	0.0226 (7)	0.0221 (8)	−0.0003 (6)	0.0016 (6)	−0.0034 (7)
N3	0.0188 (7)	0.0180 (7)	0.0224 (8)	−0.0013 (5)	0.0005 (6)	0.0008 (6)
C1	0.0303 (9)	0.0197 (9)	0.0251 (9)	0.0060 (7)	0.0049 (7)	0.0054 (8)
C2	0.0357 (11)	0.0317 (10)	0.0386 (12)	0.0123 (8)	0.0153 (9)	0.0113 (9)
C3	0.0226 (9)	0.0422 (13)	0.0560 (15)	0.0027 (9)	0.0127 (9)	0.0221 (11)
C4	0.0235 (10)	0.0342 (11)	0.0474 (14)	−0.0054 (8)	−0.0060 (9)	0.0125 (10)
C5	0.0239 (9)	0.0234 (9)	0.0290 (10)	0.0000 (7)	−0.0017 (8)	0.0070 (8)
C6	0.0206 (9)	0.0163 (9)	0.0243 (9)	0.0042 (6)	0.0028 (7)	0.0052 (7)
C7	0.0207 (8)	0.0157 (8)	0.0150 (8)	0.0016 (6)	−0.0026 (6)	0.0016 (7)
C8	0.0232 (8)	0.0168 (8)	0.0299 (10)	0.0042 (7)	0.0048 (7)	0.0043 (7)
C9	0.0179 (8)	0.0157 (8)	0.0203 (9)	−0.0031 (6)	0.0001 (7)	−0.0007 (7)
C10	0.0203 (9)	0.0171 (8)	0.0230 (10)	−0.0046 (6)	−0.0050 (7)	0.0011 (7)
C11	0.0256 (9)	0.0255 (9)	0.0203 (10)	−0.0056 (7)	−0.0047 (7)	0.0038 (8)
C12	0.0219 (9)	0.0282 (9)	0.0192 (10)	−0.0072 (7)	0.0004 (6)	−0.0042 (8)
C13	0.0269 (9)	0.0276 (10)	0.0294 (11)	0.0031 (7)	−0.0037 (8)	0.0038 (8)
C14	0.0322 (11)	0.0547 (13)	0.0220 (11)	0.0013 (9)	0.0024 (8)	−0.0055 (9)

### Geometric parameters (Å, °)

**Table:**

S1—C9	1.7646 (17)	C4—H4	0.9300
S1—C8	1.7947 (17)	C5—C6	1.385 (3)
Cl1—C1	1.742 (2)	C5—H5	0.9300
O1—C7	1.2252 (19)	C7—C8	1.516 (2)
N1—C7	1.351 (2)	C8—H8A	0.9700
N1—C6	1.416 (2)	C8—H8B	0.9700
N1—H1	0.874 (9)	C10—C11	1.385 (3)
N2—C9	1.327 (2)	C10—C13	1.502 (3)
N2—C12	1.348 (2)	C11—C12	1.387 (2)
N3—C9	1.341 (2)	C11—H11	0.9300
N3—C10	1.344 (2)	C12—C14	1.497 (3)
C1—C6	1.387 (2)	C13—H13A	0.9600
C1—C2	1.388 (3)	C13—H13B	0.9600
C2—C3	1.365 (3)	C13—H13C	0.9600
C2—H2	0.9300	C14—H14A	0.9600
C3—C4	1.383 (3)	C14—H14B	0.9600
C3—H3	0.9300	C14—H14C	0.9600
C4—C5	1.392 (3)		
			
C9—S1—C8	100.53 (8)	S1—C8—H8A	108.7
C7—N1—C6	124.06 (14)	C7—C8—H8B	108.7
C7—N1—H1	117.9 (13)	S1—C8—H8B	108.7
C6—N1—H1	117.4 (13)	H8A—C8—H8B	107.6
C9—N2—C12	115.58 (14)	N2—C9—N3	128.40 (15)
C9—N3—C10	115.01 (14)	N2—C9—S1	118.89 (12)
C6—C1—C2	121.14 (18)	N3—C9—S1	112.71 (12)
C6—C1—Cl1	119.72 (14)	N3—C10—C11	121.67 (15)
C2—C1—Cl1	119.13 (16)	N3—C10—C13	116.00 (15)
C3—C2—C1	119.6 (2)	C11—C10—C13	122.32 (16)
C3—C2—H2	120.2	C10—C11—C12	118.23 (16)
C1—C2—H2	120.2	C10—C11—H11	120.9
C2—C3—C4	120.55 (19)	C12—C11—H11	120.9
C2—C3—H3	119.7	N2—C12—C11	121.10 (16)
C4—C3—H3	119.7	N2—C12—C14	116.10 (16)
C3—C4—C5	119.7 (2)	C11—C12—C14	122.79 (17)
C3—C4—H4	120.2	C10—C13—H13A	109.5
C5—C4—H4	120.2	C10—C13—H13B	109.5
C6—C5—C4	120.50 (19)	H13A—C13—H13B	109.5
C6—C5—H5	119.8	C10—C13—H13C	109.5
C4—C5—H5	119.8	H13A—C13—H13C	109.5
C5—C6—C1	118.53 (16)	H13B—C13—H13C	109.5
C5—C6—N1	121.47 (16)	C12—C14—H14A	109.5
C1—C6—N1	120.00 (16)	C12—C14—H14B	109.5
O1—C7—N1	123.63 (14)	H14A—C14—H14B	109.5
O1—C7—C8	123.26 (14)	C12—C14—H14C	109.5
N1—C7—C8	113.10 (14)	H14A—C14—H14C	109.5
C7—C8—S1	114.09 (12)	H14B—C14—H14C	109.5
C7—C8—H8A	108.7		
			
C6—C1—C2—C3	1.2 (3)	N1—C7—C8—S1	171.73 (12)
Cl1—C1—C2—C3	−178.03 (15)	C9—S1—C8—C7	−68.05 (14)
C1—C2—C3—C4	−0.8 (3)	C12—N2—C9—N3	−0.4 (3)
C2—C3—C4—C5	−0.3 (3)	C12—N2—C9—S1	178.49 (12)
C3—C4—C5—C6	1.1 (3)	C10—N3—C9—N2	0.4 (2)
C4—C5—C6—C1	−0.7 (3)	C10—N3—C9—S1	−178.57 (11)
C4—C5—C6—N1	179.97 (16)	C8—S1—C9—N2	0.91 (15)
C2—C1—C6—C5	−0.4 (3)	C8—S1—C9—N3	179.98 (11)
Cl1—C1—C6—C5	178.80 (13)	C9—N3—C10—C11	0.4 (2)
C2—C1—C6—N1	178.91 (16)	C9—N3—C10—C13	−178.32 (14)
Cl1—C1—C6—N1	−1.9 (2)	N3—C10—C11—C12	−1.0 (2)
C7—N1—C6—C5	−48.9 (2)	C13—C10—C11—C12	177.59 (16)
C7—N1—C6—C1	131.82 (18)	C9—N2—C12—C11	−0.3 (2)
C6—N1—C7—O1	3.2 (3)	C9—N2—C12—C14	−179.39 (16)
C6—N1—C7—C8	−177.61 (15)	C10—C11—C12—N2	1.0 (2)
O1—C7—C8—S1	−9.1 (2)	C10—C11—C12—C14	179.99 (17)

### Hydrogen-bond geometry (Å, °)

**Table:**

*D*—H···*A*	*D*—H	H···*A*	*D*···*A*	*D*—H···*A*
N1—H1···O1^i^	0.87 (1)	2.05 (1)	2.8414 (18)	150 (2)
C2—H2···O1^ii^	0.93	2.46	3.213 (2)	138
C8—H8A···Cg1^i^	0.97	2.92	3.832 (2)	157
C13—H13B···Cg1^iii^	0.96	2.99	3.592 (2)	122

Symmetry codes: (i) *x*, *y*+1, *z*; (ii) −*x*+1, −*y*+2, *z*+1/2; (iii) *x*, *y*−1, *z*.

## References

[bb1] Flack, H. D. (1983). *Acta Cryst.* A**39**, 876–881.

[bb2] Gao, Y., Liang, D., Gao, L.-X., Fang, G.-J. & Wang, W. (2007). *Acta Cryst.* E**63**, o4854.

[bb3] Huynh, M. H. V., Hiskey, M. A. & Archuleta, J. G. (2005). *Angew Chem. Int. Ed.* **44**, 737–739. 10.1002/anie.200461758 15612069

[bb4] Molecular Structure Corporation & Rigaku (1999). *CrystalClear* MSC, The Woodlands, Texas, USA, and Rigaku Corporation, Tokyo, Japan.

[bb5] Sheldrick, G. M. (1996). *SADABS* University of Göttingen, Germany.

[bb6] Sheldrick, G. M. (2008). *Acta Cryst.* A**64**, 112–122. 10.1107/S0108767307043930 18156677

[bb7] Ye, C. F., Gao, H. X. & Boatz, J. A. (2006). *Angew. Chem. Int. Ed.* **45**, 7262–7265. 10.1002/anie.200602778 16969890

